# Impact of maternal and child dental anxiety on oral health-related quality of life of 5–6-year-old preschool children

**DOI:** 10.1186/s12955-020-01565-z

**Published:** 2020-09-29

**Authors:** Rashidah Esa, Marhazlinda Jamaludin, Zamros Yuzadi Mohd Yusof

**Affiliations:** 1 Department of Dental Public Health, Faculty of Dentistry, MAHSA University, Bandar Saujana Putra, 42610 Jenjarom, Selangor Malaysia; 2 Department of Community Oral Health and Clinical Prevention, Faculty of Dentistry, University of Malaya, 50603 Kuala Lumpur, Malaysia; 3 Community Oral Health Research Group, Faculty of Dentistry, University of Malaya, 50603 Kuala Lumpur, Malaysia

**Keywords:** Dental anxiety, Oral health related quality of life, Dental caries, Children, Structural equation modelling

## Abstract

**Background:**

There is a lack of evidence with regards to the association between both maternal and child dental anxiety and the mother’s perception of her child’s oral health-related quality of life (COHRQoL). The aim of this study was to investigate the association of maternal and child dental anxiety with COHRQoL and the effect of demographic factors as moderators in this relationship. In addition, the association between child’s dental caries experience and the COHRQoL was also assessed.

**Methods:**

A cross-sectional study was conducted involving 1150, 5–6 year-old preschool children in Selangor, Malaysia. Mothers answered a questionnaire on socio-economic status, the Malay-Modified Dental Anxiety Scale to assess maternal dental anxiety, and the Malay-Early Childhood Oral Health Impact Scale to assess COHRQoL. Child's dental anxiety was assessed using the Malay-Modified Child Dental Anxiety Scale via a face-to-face interview prior to oral examination to assess dental caries. Data were analysed using structural equation modelling to assess the relationship between maternal and child dental anxiety and COHRQoL.

**Results:**

Overall, complete data on 842 mother–child dyads were analysed. The mean scores of total ECOHIS, the child impacts section (CIS), and the family impacts section (FIS) were 17.7 (SD = 4.9), 12.6 (SD = 3.7), and 5.1 (SD = 1.9), respectively. The mean dental anxiety scores for mothers and children were 11.8 (SD = 4.5) and 16.9 (SD = 4.3), respectively. Maternal dental anxiety was associated with the CIS (*b* = 0.08, *p* < 0.001), the FIS (*b* = 0.01, *p* = 0.001), and the total ECOHIS (*b* = 0.14, *p* < 0.001). Maternal education level, income, urban/rural location, and kindergarten type were moderators to the relationship. In addition, there was also a significant relationship between child’s dental caries experience and COHRQoL (*p* < 0.001).

**Conclusions:**

Maternal dental anxiety and child’s dental caries experience have significantly impacted the COHRQoL, the CIS, and the FIS domains. Demographic factors such as maternal education, income, urban/rural location, and kindergarten type acted as moderators that can strengthen or weaken the relationship between maternal dental anxiety and COHRQoL of 5–6-year-old preschool children.

## Background

The term dental anxiety or fear has often been used synonymously in the literature. Dental anxiety can be described as a vague, unpleasant feeling accompanied by a premonition that something undesirable is about to happen, whereas dental fear is the anticipation of threat or harm elicited by an identifiable source [1–3]. Dental anxiety is a common problem worldwide where less than 20% of adults suffer from higher levels of dental anxiety and have had their lives affected [4, 5].

In Malaysia, the oral health programme for antenatal mothers was introduced in the early 1970s; however, only 39.2% of pregnant mothers utilised this service [6]. An earlier study revealed that among the barriers cited were ‘being scared of treatment’ and ‘fear of seeing the dentist’ [7]. Similarly, about 15% of the world’s population avoids dental care to some extent due to fear [8–10]. Higher level of anxiety is not only associated with poorer oral health status but also causes psychosocial problems in children and adults [6, 11–14]. While assisting highly anxious patients overcome their fear of dental treatment poses its own set of challenges, its success will no doubt result in the improvement of their oral health status, quality of life, and well-being [15, 16].

Besides dental anxiety, quality of life is also important as it helps us to understand the impact of dental problems and their treatment on patients’ well-being [16–19]. The impact of oral health on quality of life is assessed using the oral health-related quality of life (OHRQoL) instruments [20]. Such instruments aim to measure the perceived impact of oral health on daily performances, psychosocial well-being, and social life. Assessing such impact in young children is essential because children with poor oral health will face future consequences not only on the development of permanent teeth, but also on their physical growth, weight, self-esteem, social life, and school achievement [21, 22]. Furthermore, as parents look after their young children, their dental problems frequently have extended consequences on the parents and family members who live with the child [23, 24].

A meta-analysis had reported a relationship between parental and child dental fear in children aged 8 years and below [25]. However, the strength of the relationship was dependent on the context of the dental visit and the types of measures used for assessing dental anxiety. At the same time, children whose mothers had moderate or high levels of dental anxiety were more likely to have untreated dental caries than children whose mothers had low dental anxiety [17, 26, 27]. In addition, many studies had reported that maternal dental anxiety, socioeconomic status, and oral health behaviours were also associated with poorer child’s OHRQoL (COHRQoL) [17, 18, 28, 29]. On the other hand, child dental anxiety was associated with child’s dental caries [30], and child’s dental caries was associated with COHRQoL [22]. However, there’s a lack of evidence with regards to the association between both maternal and child dental anxiety and the mother’s perception of her COHRQoL, especially in the local population. As a result, it was hypothesised that maternal and child dental anxiety have a significant impact on COHRQoL. Findings from this study will be important in designing effective interventions to alleviate dental anxiety in mother and child within the family and to improve the COHRQoL.

Therefore, the aim of this study was to investigate the association of maternal and child dental anxiety with COHRQoL and the effect of demographic factors as moderators in this relationship. In addition, the association between child’s dental caries experience and the COHRQoL was also assessed.

## Methods

This study, conducted in accordance with the 1964 Declaration of Helsinki, was approved by the Medical Ethics Committee, Faculty of Dentistry, University of Malaya [Reference: DF OP0809/0030(L)]. Permission to conduct the study was obtained from the Ministry of Education, Selangor State Education Department, kindergarten teachers, and parents of the children. Informed written consent was obtained from all participants included in the study prior to data collection.

This cross-sectional study was conducted in the Petaling (urban) and Hulu Langat (rural) districts in Selangor, Malaysia. Sample size calculation was based on the following parameters: design effect of 2.7 from a pilot study to account for the cluster sampling design, COHRQoL prevalence of 71.0% from a local study involving 5–6-year-old children [31], and a 35% non-response rate resulting in a sample size of 1150 mother–child dyads. In terms of sample selection, two districts representing urban and rural areas were randomly chosen. In each district, kindergartens were stratified into private and public (government-run). Based on the student enrolment ratio, a total of 920 children were required from urban areas which included 230 and 690 children from public and private kindergartens, respectively. In rural areas, only 230 children were required which comprised of 115 children each from public and private kindergartens. A random number generator was used to choose the respective kindergartens.

The inclusion criteria were mothers who can speak and write in the Malay language and live with their 5–6-year-old children. Only healthy children were included. Children with chronic medical problems, on long-term medications, or who suffer from either physical or learning disabilities were excluded.

The questionnaire used in this study comprised of the mother’s and child’s socio-demographic information, the Malay version of the Modified Dental Anxiety Scale (Malay-MDAS) [32], the Malay version of the Modified Child Dental Anxiety Scale faces version (Malay-MCDAS_f_) [33]_,_ and the Malay version of the Early Childhood Oral Health Impact Scale (Malay-ECOHIS) [31]. Family monthly incomes were categorised into low (MYR < 1500), moderate (MYR 1500 to 3500), and high income (> MYR 3500) in line with the minimum wage in Malaysia during the data collection period [34].

The Malay-MDAS was translated from the English MDAS [35, 36] to assess maternal dental anxiety. The Malay-MCDAS_f_ was translated from the English MCDAS_f_ [37] to assess child’s dental anxiety. The English MCDAS_f_ was developed from the MCDAS [38, 39] where a faces rating scale was added to assess dental anxiety in young children. The Malay-ECOHIS was translated from the English ECOHIS that was developed and validated in the USA among 2–5-year-old children and their families [40]. In this study, the Malay-ECOHIS was used as a proxy-reported measure to assess oral impacts among preschool children in the Malaysian setting. It was validated for children up to 6 years old and therefore its use in this study was justified.

The Malay-MDAS has similar response options to the English MDAS, and the additional fifth item assesses anxiety when receiving a local anaesthetic injection. The response options are standardised to a five-point scale from “not anxious” to “extremely anxious” with the total score ranging from 5 to 25. A score of 19 or more is regarded as extremely dentally anxious, equivalent to dental phobia [35]. In our study, higher Malay-MDAS scores indicated higher maternal dental anxiety levels.

The Malay-MCDAS_f_ is a child-reported measure consisting of six questions about visiting the dentist and dental procedures. Each question is rated on a five-point rating scale ranging from “relaxed or not worried” to “very worried”. Many studies have used either the mean or the median score as a cut-off point for dentally anxious [37, 41–46]; however, the use of this cut-off point may introduce bias in the conclusions, as the proportion of the population categorised as dentally anxious has been predetermined. In this study, the Malay-MCDAS_f_ scores ranged from 6 to 30 points where higher scores indicate higher child dental anxiety levels. The children’s anxiety scores were categorised as low (6 to 10 points), moderate (11 to 20 points), or high (24 to 26 points) based on previous studies [41–43]. However, the child dental anxiety scores were used as a continuous variable in the Structural Equation Modelling (SEM) analysis.

The Malay-ECOHIS has 13 items in two main parts: the child impacts section (CIS) and the family impacts section (FIS). The CIS has 4 domains with 9 items: child symptoms (1 item), child function (4 items), child psychology (2 items), and child self-image/social interaction (2 items). The FIS has 2 domains with 4 items: parental distress (2 items) and family function (2 items). Each item is rated using five response options: 0 = never, 1 = hardly ever, 2 = occasionally, 3 = often, 4 = very often, and 5 = don’t know. The total score was obtained by summing the scores of the 13 items which ranges from 0 to 52 (0 to 36 for the CIS, 0 to 16 for the FIS). Items answered with ‘don’t know’ responses were recoded as missing values. For up to two ‘don’t know’ responses in the CIS and one in the FIS, the missing values were replaced by the mean score of the remaining items for that particular section. We excluded participants with three or more ‘don’t know’ responses in the CIS or two or more ‘don’t know’ responses in the FIS following the method of data scoring proposed in the original version [40]. In this study, higher Malay-ECOHIS scores indicated higher oral impacts and poorer OHRQoL. For the category of impact, response options ‘never’ or ‘hardly ever’ were categorised into ‘no impact’ while ‘occasionally’ to ‘very often’ were categorised into ‘with impact’.

The self-administered questionnaire, patient information sheet, and consent form were distributed to the mothers by the teachers. The mothers returned the signed consent form and the completed parental questionnaire before the children were interviewed and underwent oral examination. Only children whose mothers provided written consent were included in the study. Children’s dental anxiety was assessed via individual face-to-face interviews. Two trained dental surgery assistants and the main author (RE) conducted the interviews. The children were briefed on the response options of the Malay-MCDAS_f_ faces scale before they were interviewed.

In terms of oral examination, four dental examiners underwent calibration in diagnosing caries at dental school. Dental caries of the primary teeth was assessed as decayed, missing, or filled teeth (dmft). Caries was diagnosed as clinically detectable lesions in dentine (D3 level) without the use of a probe. The four dental examiners examined 20 children for caries and their findings were compared with that of a gold standard who was a paediatric dental specialist. Re-examination was conducted after one week. Kappa statistics were used to assess the inter- and intra-examiner reliability for caries in primary teeth. The results showed that the inter-examiner Kappa values for dental caries ranged from 0.76 to 0.79, indicating good reliability while the intra-examiner Kappa values ranged from 0.65 to 1, indicating moderate to very good reliability [47]. In the actual study, oral examinations were performed using a plane mouth mirror under natural light in the kindergarten. Following the oral examination, mothers were given brief advice on their child’s dental caries status. Children with caries who required treatment were referred to a nearby dental clinic.

Data were analysed using the Statistical Package for Social Sciences (SPSS) Version 22 software (SPSS Inc., Chicago, IL, USA). Descriptive statistics (mean and standard deviation, frequency and percentage) were used to describe the socio-demographic data of the participants, the MDAS, the MCDAS_f_, and the ECOHIS domains. Next, Structural Equation Modelling (SEM) was designed to determine the impact of maternal and child dental anxiety, and child dental caries on COHRQoL (Model 1). Model 2 was designed to test the impact of maternal and child dental anxiety, and child dental caries on COHRQoL in subconstructs (CIS and FIS) in order to examine which subconstruct will have more impact on COHRQoL. In addition, Model 3 was only to test the impact of moderators (demographic factors) on the relationship of maternal dental anxiety → COHRQoL as only maternal dental anxiety showed significant impact towards COHRQoL.

In this study, the intra-cluster correlation was not calculated in the sample due to the fact that the analysis using SEM has the flexibility to handle data involving complex sampling issues with cluster samples in multistage sampling [48]. Furthermore, the sample size of the study was large enough that the assumption of normality of data was achieved.

The statistical package used was Analysis of Moment Structures Version 22 (AMOS V 22) by using Maximum Likelihood Estimation. The model fitness indices accessed indices of (1) Absolute fit comprised of Goodness of Fit Index (GFI) and Root Mean Square of Error Approximation (RMSEA), (2) Incremental fit comprised of Comparative Fit Index (CFI), and (3) Parsimonious fit comprised of chi square/df [49]. The model testing for the relationship between domains was set at the significant level of *p* < 0.05.

## Results

Of the 1150 questionnaires distributed to 24 kindergartens, a total of 873 mothers returned the questionnaire (75.9% response rate). Three children refused to be examined and were excluded while another 28 questionnaires were incomplete. As a result, a total of 842 complete mother–child dyads were included in the data analysis.

Table 1 shows the socio-demographic and clinical characteristics of the participants. More than half of the children were girls (52.3%) and of Malay ethnicity. The mean age was 5.6 (SD = 0.5) years. Most of the children attended private kindergartens while the majority of kindergartens were located in urban areas. Almost half of the mothers had secondary school education, and over two-fifths had a monthly income of MYR1500-3500 (moderate income level). More than two-thirds of the children had caries with a mean overall dmft score of 4.3 (SD = 4.6).

**Table 1:** Socio-demographic characteristics of the participants including clinical characteristics of children aged 5–6 years (n = 842)

	Mother n (%)	Child n (%)
Mean age/year (SD)	37.2 (12.7)	5.6 (0.5)
Age group/year		
5	–	363 (43.1)
6		479 (56.9)
Gender		
Boy	–	402 (47.7)
Girl		440 (52.3)
Ethnicity		
Malay	768 (91.2)	776 (92.1)
Chinese	14 (1.7)	14 (1.7)
Indian	29 (3.4)	31 (3.7)
Others	31 (3.7)	21 (2.5)
Level of education^a^		
No formal schooling/primary school	28 (3.4)	–
Secondary school	415 (49.3)	
College or university	382 (45.4)	
Monthly income (MYR)^b^		
Low < 1500	162 (19.2)	–
Moderate 1500–3500	347 (41.2)	
High > 3500	318 (37.8)	
Location of kindergarten		
Rural	–	248 (29.5)
Urban		594 (70.5)
Type of kindergarten		
Public/government	–	310 (36.8)
Private		532 (63.2)
Caries status		
Caries-free	–	270 (32.1)
With caries		572 (67.9)
Caries experience		
Mean dmft (SD)	–	4.3 (4.6)
Mean dt (SD)		4.0 (4.5)
Mean mt (SD)		0.1 (0.4)
Mean ft (SD)		0.2 (0.7)

*MYR* Malaysian Ringgit

^a^Missing values for level of education = 17 (2%)

^b^Missing values for monthly income = 15 (1.8%)

Table 2 shows the summary of total ECOHIS, CIS, FIS, child dental anxiety (MCDAS_f_), and maternal dental anxiety (MDAS) scores. The mean scores of total ECOHIS, CIS, and FIS subscales were quite low. The mean scores for all 6 domains of ECOHIS were skewed towards the minimum score range. The 6-item MCDAS_f_ scores ranged from 6 to 30 with a mean score of 16.9 (SD = 4.3). More than half of the children reported having high anxiety levels. For the mothers, the 5-item MDAS scores ranged from 5 to 25 with a mean score of 11.8 (SD = 4.5). A large majority of the mothers had low or moderate dental anxiety.

**Table 2:** Summary data on total ECOHIS, the CIS, FIS, child dental anxiety (MCDAS_f_), and maternal dental anxiety (MDAS) (n = 842)

	Possible minimum and maximum score range	Score range	Mean (SD)	Frequency (%)
ECOHIS (total)^a^	13–52	13–38	17.7 (4.9)	
CIS	9–36	9–27	12.6 (3.7)	–
FIS	4–16	4–20	5.1 (1.9)	
ECOHIS domain:				
Child symptoms (1 item)	1–4	1–4	1.7 (0.9)	
Child function (4 items)	4–16	4–12	5.3 (1.7)	
Child psychology (2 items)	2–8	2–8	3.0 (1.4)	–
Child self-image (2 items)	2–8	2–8	2.5 (1.2)	
Parent distress (2 items)^a^	2–8	2–8	2.9 (1.5)	
Family function (2 items)^b^	2–8	2–8	2.3 (0.7)	
Child dental anxiety (MCDAS_f_)	6–30	6–30	16.9 (4.3)	–
Child dental anxiety (MCDAS_f_)				
Low/moderate	–	–	–	388 (46.1)
High				454 (53.9)
Maternal dental anxiety (MDAS)	5–25	5–25	11.8 (4.5)	–
Maternal dental anxiety (MDAS)				
Low/moderate	–	–	–	769 (91.3)
High				73 (8.7)

^a^Missing values = 14 (1.4%)

^b^Missing values = 3 (0.4%)

Table 3 shows the overview of responses to the ECOHIS items and domains. More children were reported to have experienced an impact in the *child symptoms* domain (27.2%). This was followed by the *child psychology* domain with “become irritable and frustrated” (23.3%). This was followed by the *child function* domain with “difficulty in eating some foods” (15.6%) and “difficulty in pronouncing any words” (14.7%). This was followed by the *child self-image* domain. In the FIS subscale, *parent distress* was the more frequently affected domain with “parent felt guilty” (13.6%) and “parent been upset” (13.3%), followed by the *family function* domain.

**Table 3:** Overview of responses to the ECOHIS items (n = 842)

Study variable	Never n (%)	Hardly ever n (%)	Occasionally n (%)	Often n (%)	Very often n (%)
*Child symptoms (1 item)*					
Pain in the teeth, mouth or jaw	456 (54.2)	157 (18.6)	216 (25.7)	11 (1.3)	2 (0.2)
*Child function (4 items)*					
Had difficulty in drinking hot & cold beverages	682 (81.0)	79 (9.4)	76 (9.0)	5 (0.6)	–
Had difficulty in eating some Foods	583 (69.2)	128 (15.2)	112 (13.3)	18 (2.1)	1 (0.1)
Had difficulty in pronouncing any words	624 (74.1)	94 (11.2)	109 (12.9)	12 (1.4)	3 (0.4)
Missed school, day care	815 (96.8)	16 (1.9)	10 (1.2)	–	1 (0.1)
*Child psychology (2 items)*					
Had trouble sleeping	652 (77.4)	81 (9.6)	94 (11.2)	15 (1.8)	–
Became irritable and frustrated	511 (60.7)	135 (16.0)	168 (20.0)	24 (2.9)	4 (0.5)
*Child self-image (2 items)*					
Avoided smiling or laughing	687 (81.6)	78 (9.3)	73 (8.7)	4 (0.5)	–
Avoided talking	700 (83.1)	60 (7.1)	79 (9.4)	3 (0.4)	–
*Parent distress (2 items)*					
Been upset^a^	610 (72.4)	120 (14.3)	81 (9.6)	14 (1.7)	5 (0.6)
Felt guilty^a^	567 (67.3)	161 (19.1)	76 (9.0)	18 (2.1)	8 (1.0)
*Family function (2 items)*					
Taken time off^b^	762 (90.5)	44 (5.2)	31 (3.7)	1 (0.1)	1 (0.1)
Financial impact^b^	787 (93.5)	17 (2.0)	33 (3.9)	1 (0.1)	1 (0.1)

^a^Missing values = 12 (1.4%)

^b^Missing values = 3 (0.4%)

Table 4 shows the results of Structural Model Impact of maternal dental anxiety and child dental anxiety on COHRQoL (total ECOHIS), CIS, and FIS (n = 842). Maternal dental anxiety and child dental caries (dmft) showed significant impact on COHRQoL, CIS, and FIS (*p* < 0.05). Maternal dental anxiety also showed significant impact on child dental caries (*p* = 0.014). However, child dental anxiety does not have any significant direct impact on COHRQoL, CIS, FIS, or child dental caries.

**Table 4:** Path Analysis for Structural Model Impact of Mother’s Dental Anxiety, Children’s Dental Anxiety (Model 1), and Children’s Dental Caries (Model 2) on COHRQoL, Child Impact Section (CIS) and Family Impact Section (FIS) [n = 842]

Relationship	Beta estimate	S.E	C.R	*p *value
Mother’s Dental Anxiety → COHRQoL	0.14	0.03	− 5.02	< 0.001
Children’s Dental Anxiety → COHRQoL	0.00	0.03	0.01	0.998
Children’s Dental Caries → COHRQoL	0.04	0.01	− 6.74	< 0.001
Mother’s Dental Anxiety → CIS	0.08	0.02	− 4.71	< 0.001
Children’s Dental Anxiety → CIS	0.01	0.02	− 0.30	0.765
Children’s Dental Caries → CIS	0.01	0.01	− 3.23	0.001
Mother’s Dental Anxiety → FIS	0.01	0.01	− 3.18	0.001
Children’s Dental Anxiety → FIS	0.01	0.01	− 0.38	0.704
Children’s Dental Caries → FIS	0.10	0.01	− 4.72	< 0.001

**Fig. 1:**
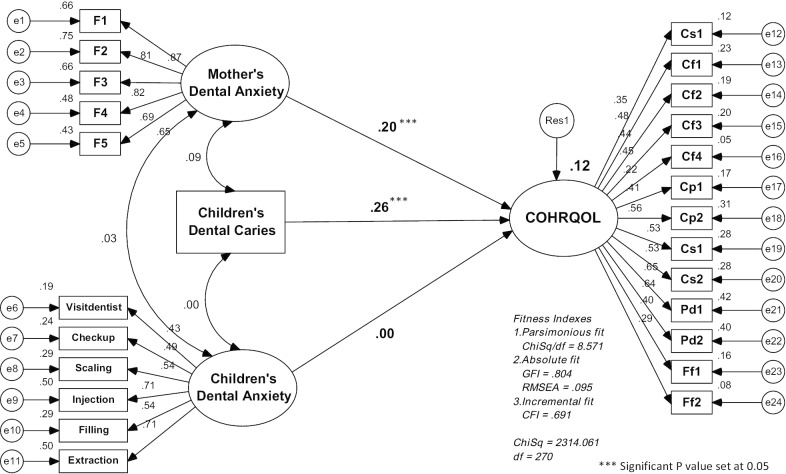
Standardized structural model impact of Mother’s Dental Anxiety, Children’s Dental Anxiety, and Children’s Dental Caries on COHRQoL (n = 842)

Figure 1 shows the Standardised Structural Model Impact of maternal and child’s dental anxiety on COHRQoL. Children’s OHRQoL was dependent on maternal dental anxiety and child’s dental caries by 12.0%. The items for each domain have factor loading ranging from 0.22 to 0.87. Although some literature states that items with loading below 0.5 should be discarded from the model, they were maintained in this study as there was strong evidence that the items were important in this structural model [31]. Since all the items were retained in the model, this influenced the model fit indices (the model fitness almost approaching the cut off point; chisq/df < 5.0, GFI > 0.90, RMSEA < 0.10, CFI > 0.90). According to Browne and Cudeck [50], the values of RMSEA ranging from 0.08 to 0.10 are considered as a mediocre fit. Only RMSEA (RMSEA = 0.095) fulfilled the model fit indices. The correlation between mother’s dental anxiety and children’s dental anxiety was very low (r = 0.03).

Table 5 shows the moderator test for the demographic influence on the maternal dental anxiety and COHRQoL relationship. Since there was a significant effect of maternal dental anxiety on COHRQoL in the Structural Equation Model, this study proceeded to investigate demographic factors as a moderator in this relationship. The demographic variables selected were mother’s education level, household income, location of kindergarten, and type of kindergarten. The maternal dental anxiety—COHRQoL model was constraining with covariance which resulted in a chi square difference above 3.84. These results show that all the demographic variables selected can act as moderators in the maternal dental anxiety—COHRQoL model.

**Table 5:** Demographic effect on the Maternal Dental Anxiety and COHRQoL relationship (Model 3) (n = 842)

Variable tested	Χ^2^ value		
	Constrained model (A)	Unconstrained model (B)	Difference (A − B)
Education			
Low	1172.98	901.49	271.49
High	1204.81	909.54	295.27
Income			
Low to moderate	1245.52	942.86	302.66
High	1216.55	949.61	266.94
Location			
Rural	798.59	650.49	148.10
Urban	1663.31	1201.90	461.41
Type			
Government/public	756.81	556.21	200.60
Private	1659.44	1233.40	426.04

Constrained model; df = 135, Unconstrained model; df = 134

Education Level; Low level comprises of No formal education, Primary school and Secondary school, High level comprises of College and university

Income Level; Low to moderate (income less than MYR3500), High (income MYR3500 and above)

Table 6 shows the effect of demographics on the maternal dental anxiety and COHRQoL relationship. All the levels in demographic groups were significant in the model (*p* < 0.05). Maternal dental anxiety with high education level shows better COHRQoL (standardized beta estimate = 0.29, *p* < 0.001). Similarly, maternal dental anxiety with high income level shows better COHRQoL (standardized beta estimate = 0.23, *p* < 0.001).

**Table 6:** Demographic effect on Maternal Dental Anxiety and COHRQoL relationship (Model 3) (n = 842)

Demographic effect	Standardised beta estimate	*p *value	
Education	Low	0.13	0.026
	High	0.29	< 0.001
Income	Low to moderate	0.19	< 0.001
	High	0.23	< 0.001
Location	Rural	0.22	0.004
	Urban	0.23	< 0.001
Type	Government/public	0.19	0.006
	Private	0.26	< 0.001

## Discussion

This study investigated the impact of maternal and child dental anxiety on the OHRQoL of 5–6-year-old preschool children. The role of demographic factors and child’s dental caries experience with the COHRQoL were also evaluated. Structural equation models were designed to inspect the relationships between maternal dental anxiety, child dental anxiety, child dental caries, and COHRQoL and its domains. In addition, the role of demographic factors as moderators was also determined.

Mothers are the main caregivers responsible for child-rearing and transmitting health related behaviours to their children [18, 29]. A large sample of preschool children where the majority of parents were young mothers of Malay ethnicity aged less than 40 years participated in this study. The sample demographic background was almost similar to the Malaysian population strata [51]. Also, there was a slight discrepancy between the ethnicity of mothers and children, the reason being the ethnicity of the children followed that of the father.

Past studies had reported that higher paternal or maternal dental anxiety was associated with poorer OHRQoL of their preschool children [17, 29, 52]. This study also found that maternal dental anxiety and child dental caries (dmft) showed significant impact on COHRQoL (total ECOHIS). Higher MDAS scores would highly influence how the mother perceives the impact of child oral health on the total ECOHIS scores. In other words, mothers who are more anxious would “rate” their children’s OHRQoL poorly. Although some of the items for each domain of the ECOHIS have low factor loadings (below 0.5), these 13 items of the ECOHIS were retained in the model as this could be due to the very low mean scores of total ECOHIS subscales in this sample. The mean scores for all 6 domains of ECOHIS were also skewed towards the minimum score range. Additionally, for the category of impact response options ‘never’ or ‘hardly ever’ were categorised into ‘no impact’. The scores for ‘no impact’ ranged from 72.8% (child symptom) to 98.7% (missed school or day care). The variation within these items would be attenuated and hence, unlikely to produce a good model fit. Future studies using qualitative methods are recommended to review the items as social and cultural variations exist within this diverse population [6, 53].

Nevertheless, these findings may still be of importance because the impacts would directly influence the child’s daily activities, psychosocial well-being, as well as the family’s general functioning. Furthermore, the magnitude of association will tend to be greater as the mother’s dental anxiety level gets higher.

In this study, anxious mothers were significantly associated with poorer COHRQoL in both the CIS and FIS domains. However, these findings were different from that of Goettems et al. [17]. The latter reported that the impact of maternal dental anxiety was mainly on the *parent distress* domain. This difference could be due to the geographical and cultural variations between the samples in the two studies. In Goettems’ study, only maternal dental anxiety was assessed and the ECOHIS was assessed with children aged 2–5 years. Furthermore, the four-item Corah’s DAS scale to assess maternal dental anxiety was used in that study [17].

In addition, child’s dental caries was found to be associated with maternal dental anxiety and COHRQoL. The children’s overall dmft score was 4.3 of which 93.0% comprised of decayed teeth. This finding was not unexpected as children with decayed and missing teeth tend to complain of pain as well as difficulties in eating and pronouncing words. The mean dmft score in this study was almost similar to the mean dmft score of 4.6 among 5 year-old preschool children in a recent national oral health survey [54]. Other studies also found a similar finding where untreated dental caries had affected the quality of life of the children [19, 22]. Buldur and Guvendi [18] found an indirect relationship between parental dental anxiety, dental caries, and COHRQoL. However, Low et al., [55] reported that children may not complain of pain from decayed teeth but may be affected by other impacts related to oral function.

In the path analysis model for the influence of demographic factors on maternal dental anxiety and COHRQoL, it was found that all four factors acted as moderators to the relationship. These four factors include education, family income, location, and type of kindergarten. Mothers with higher education and family income have better perception on the COHRQoL. It could be that mothers with different education levels have different perceptions of how their children’s oral health has impacted their daily life and well-being. The reason could be that families with higher incomes would be less likely to suffer financial impact when they bring their child to a dentist for dental treatment than families with lower incomes. Also, families with higher incomes tend to have children with better oral health [56]. Subsequently, their parents would be less likely to take time off work because of their child’s dental problems. These findings were also supported by similar studies elsewhere [17–19, 57, 58]. Sending a child to private kindergartens which are mainly situated in urban areas is also related to higher education and income. Similarly, an earlier local study also found that urban–rural dichotomy also acted as a moderator for the relationship between dental caries and dental fear among adolescents [59].

In contrast, we found that child dental anxiety does not have any significant direct impact on COHRQoL, CIS, FIS, or child dental caries. A possible reason for this could be that the decision to bring the child to visit the dentist for a check-up or preventive care is decided mainly by the mother or guardian regardless of the child’s dental anxiety level. In addition, the kindergartens in Malaysia receive an annual visit from the school dental team where a dental therapist will conduct an oral examination, do preventive treatment, and deliver oral health education to the children. As a result, the children would still benefit from the preventive care and oral health education given by the school dental team regardless of whether they were dentally anxious or not.

A high proportion of the preschool children in this study reported high anxiety levels. In contrast, the majority of the mothers reported low or moderate dental anxiety levels. Only 9.0% of mothers had high anxiety levels. The correlation between mother’s dental anxiety and children’s dental anxiety was found to be very low in this study. Few studies had reported similar findings but the majority of studies confirmed a relationship between maternal and child dental anxiety especially in children aged 8 years and below [17, 18, 25, 29, 52].

The strength of this study is employing SEM and determining the moderator effect on the relationship between maternal dental anxiety and COHRQoL. However, the development and testing of a complex multi-model needs to be explored further to include other related variables affecting this relationship.

This study had several limitations. First, the use of the Malay-ECOHIS relied on the mothers’ perceptions of their child’s oral health and its impacts on the child and family. As a result, different mothers had different perceptions of their child’s oral health and these might be influenced, among others, by the mother–child relationship, mother’s personalities, family dynamics, and the socio-environment of the family. It was probable that mothers with dental anxiety would be more likely to rate their COHRQoL poorly than mothers with no dental anxiety. This possibility could be true especially when no association was found between child dental anxiety and COHRQoL in this study. Future studies should consider the validity of proxy reports on the COHRQoL by mothers including undertaking a qualitative study involving both the mothers and children. Cohort studies to address the limitations of a cross-sectional study design in inferring causation between maternal dental anxiety and COHRQoL are also recommended [60]. Second, paternal dental anxiety was not assessed in this study. This was because in Malaysian culture, mothers play an important role in the upbringing of the child and tend to be closer to the child than fathers. Therefore, mothers would be regarded as a better proxy than fathers to assess COHRQoL. Future studies should also look into parental dental anxiety and its association with COHRQoL.

## Conclusions

Maternal dental anxiety and child’s dental caries experience have significantly impacted the COHRQoL, the CIS, and FIS domains of 5–6-year-old preschool children. In addition, demographic factors such as education, income, urban/rural location, and kindergarten type acted as moderators that modify the strengths of the relationship between maternal dental anxiety and COHRQoL.

## Data Availability

The datasets used and/or analysed during the current study are available from the corresponding author on reasonable request.

## Abbreviations

CIS: Child impacts section
COHRQoL: Child’s oral health-related quality of life
FIS: Family impacts section
Malay-ECOHIS: Malay version of Early Childhood Oral Health Impact Scale
Malay-MCDAS_f_: Malay version of Modified Child Dental Anxiety Scale faces version
Malay-MDAS: Malay version of Modified Dental Anxiety Scale
OHRQoL: Oral health-related quality of life
